# QTL Mapping of Fusarium Head Blight and Correlated Agromorphological Traits in an Elite Barley Cultivar Rasmusson

**DOI:** 10.3389/fpls.2018.01260

**Published:** 2018-08-28

**Authors:** Yadong Huang, Matthew Haas, Shane Heinen, Brian J. Steffenson, Kevin P. Smith, Gary J. Muehlbauer

**Affiliations:** ^1^Department of Agronomy and Plant Genetics, University of Minnesota, St. Paul, MN, United States; ^2^Department of Plant Pathology, University of Minnesota, St. Paul, MN, United States; ^3^Department of Plant and Microbial Biology, University of Minnesota, St. Paul, MN, United States

**Keywords:** FHB, DON, barley, elite germplasm, QTL, RIL population

## Abstract

Fusarium head blight (FHB) is an important fungal disease affecting the yield and quality of barley and other small grains. Developing and deploying resistant barley cultivars is an essential component of an integrated strategy for reducing the adverse effects of FHB. Genetic mapping studies have revealed that resistance to FHB and the accumulation of pathogen-produced mycotoxins are controlled by many quantitative trait loci (QTL) with minor effects and are highly influenced by plant morphological traits and environmental conditions. Some prior studies aimed at mapping FHB resistance have used populations derived from crossing a Swiss landrace Chevron with elite breeding lines/cultivars. Both Chevron and Peatland, a sib-line of Chevron, were used as founders in the University of Minnesota barley breeding program. To understand the native resistance that might be present in the Minnesota breeding materials, a cross of an elite cultivar with a susceptible unadapted genotype is required. Here, a mapping population of 93 recombinant inbred lines (RILs) was developed from a cross between a moderately susceptible elite cultivar ‘Rasmusson’ and a highly susceptible Japanese landrace PI 383933. This population was evaluated for FHB severity, deoxynivalenol (DON) accumulation and various agromorphological traits. Genotyping of the population was performed with the barley iSelect 9K SNP chip and 1,394 SNPs were used to develop a genetic map. FHB severity and DON accumulation were negatively correlated with plant height (HT) and spike length (SL), and positively correlated with spike density (SD). QTL analysis using composite interval mapping (CIM) identified the largest effect QTL associated with FHB and DON on the centromeric region of chromosome 7H, which was also associated with HT, SL, and SD. A minor FHB QTL and a minor DON QTL were detected on chromosome 6H and chromosome 3H, respectively, and the Rasmusson alleles contributed to resistance. The 3H DON QTL likely represents native resistance in elite germplasm as the marker haplotype of Rasmusson at this QTL is distinct from that of Chevron. This study highlights the relationship between FHB resistance/susceptibility and morphological traits and the need for breeders to account for morphology when developing FHB resistant genotypes.

## Introduction

Fusarium head blight or head scab is a major disease affecting the yield and quality of small grain cereals, especially wheat and barley worldwide. FHB is caused by multiple *Fusarium* species, but in North America *Fusarium graminearum* Schwabe is the main causal agent. During the 1990s, there were several outbreaks of FHB on wheat and barley throughout the United States that caused significant yield loss and economic hardship for many local communities (McMullen et al., 1997). From 1993 to 2001, economic losses due to FHB of barley were estimated at $485 million in the tri-state area of North Dakota, Minnesota, and South Dakota (Nganje et al., 2004). The climatic conditions conducive to FHB epidemics are a combination of prolonged high humidity and warm temperature when crops are at anthesis. Disease symptoms of FHB include tan to dark brown discoloration of spikelets and shrunken kernels. Typical signs of the pathogen on kernels include pinkish to salmon-colored masses of fungal mycelium and conidia and also blue–black perithecia. Even more important than yield loss, FHB pathogens produce mycotoxins that can contaminate the harvested grain, posing health risks to human and animals. The primary mycotoxin produced by *F*. *graminearum* is DON, a type B trichothecene. The United States Food and Drug Administration has set advisory levels for DON on grains (10 ppm) and finished wheat products (1 ppm) (FDA, 2010). The lack of FHB resistant barley genotypes makes it difficult to achieve complete control of FHB when inoculum is present and the environmental conditions are conducive for infection. An integrated field management approach is required to mitigate FHB and DON contamination in barley, which includes crop rotation with non-susceptible hosts, fungicide application, and deployment of moderately resistant cultivars (Steffenson, 2003).

Many bi-parental mapping studies have been conducted on barley to elucidate the genetic architecture of resistance to FHB and DON accumulation and to identify molecular markers that could be useful in breeding (de la Peña et al., 1999; Zhu et al., 1999; Ma et al., 2000; Dahleen et al., 2003; Mesfin et al., 2003; Hori et al., 2005; Horsley et al., 2006). These studies revealed that FHB resistance and DON accumulation are genetically complex traits. FHB resistance is a quantitative trait with low to moderate heritability. It is controlled by many minor effect QTL and is subject to extensive QTL-by-environment interactions. Moreover, many of the QTL associated with FHB resistance are coincident with QTL associated with various agronomic and morphological traits, which greatly complicate the breeding effort in elite germplasm. This finding also raises important questions about whether such coincident QTL for FHB resistance and agromorphological traits are due to tight linkage or pleiotropy, since certain agromorphological traits can provide an escape mechanism. QTL for resistance to FHB and DON accumulation have been detected on all seven barley chromosomes. de la Peña et al. (1999) identified 10 QTL for FHB resistance in a RIL population derived from a cross between Chevron and a University of Minnesota breeding line M69. These QTL explained between 0.6 and 16.0% of phenotypic variance with the larger effect QTL located in chromosome 2H bin 8 and bin 13. Ma et al. (2000) used a doubled haploid (DH) population developed from a cross of Chevron/Stander and identified nine QTL each for FHB severity and DON accumulation. Using a two-rowed by six-rowed RIL population developed from a Fredrickson/Stander cross, Mesfin et al. (2003) identified three major FHB resistance QTL all residing on chromosome 2H. Two of these three QTL were associated with a HD QTL (bin 8) or the spike row-type locus *Vrs1* (bin 10) (Komatsuda et al., 2007). Several later mapping studies using various resistance sources have detected coincidental FHB resistance QTL and morphological/developmental traits QTL in the bin 8, bin 10 and bin 13–14 regions on 2H (Lamb et al., 2009; Yu et al., 2010; Mamo and Steffenson, 2015). Exotic germplasm used as parents in previous QTL mapping studies have undesirable agronomic and morphological traits associated with FHB resistance, such as late heading and/or taller stature. However, the resolution of these studies is too low to resolve whether these unfavorable associations are due to linkage or pleiotropy. Such unfavorable associations complicate introgressing FHB resistance into elite germplasm. To overcome this difficulty, Massman et al. (2011) conducted a genome-wide association study (GWAS) using 768 advanced barley breeding lines and identified four QTL for FHB resistance and eight QTL for DON accumulation, with each QTL explaining 1–3% of the total phenotypic variation. Fewer coincident QTL were found for FHB and various agromorphological traits by Massman et al. (2011) due to the greater uniformity of germplasm used in the study.

Two of the historical founder parents in the University of Minnesota barley breeding program were Chevron (CI 1111) and Peatland (CIho 2613), which were used initially as a source of stem rust resistance back in the 1930s (Brueggeman et al., 2002). Chevron and Peatland were derived as selections from the same bulk seed lot of a landrace from Switzerland. They are very similar to each other for a number of traits including their resistance to stem rust and to some extent also to FHB (Shands, 1939; Åberg and Wiebe, 1946). Chevron was used extensively as a source of FHB resistance in breeding programs and is considered the six-rowed “gold standard” for resistance to FHB and also to KD (i.e., kernel blight) (Immer and Christensen, 1943), a disease caused primarily by *Bipolaris sorokiniana* (teleomorph *Cochliobolus sativus*), but also *Alternaria alternata* and various species of *Fusarium* as well (Wilcoxson et al., 1980; Miles et al., 1989). Given that Peatland and Chevron were used as founder parents in the Minnesota breeding program and continuous selection had been made against lines having KD (Gebhardt et al., 1992), it is possible that QTL for FHB resistance have been retained. For example, a major QTL for KD was identified on barley chromosome 6H in MNBrite, a KD resistant cultivar which was derived from Chevron through multigenerational selection for kernel brightness (Canci et al., 2003). The same QTL was confirmed by two validation populations and was also associated with FHB resistance in a later study (Canci et al., 2004). The FHB resistance loci may remain genetically cryptic if in mapping studies the other parents also possess the same resistance alleles. All of the previous mapping populations developed to study FHB resistance were made between an exotic source of FHB resistance and an advanced breeding line that was moderately susceptible. One way to shed additional light on the association of agromorphological traits and resistance would be to observe trait segregation in a cross between an exotic susceptible parent and an advanced breeding line. Additionally, to detect favorable resistance alleles present in advanced germplasm, it is best to cross to a diverse parent that is devoid of any possible resistance QTL. In the present study, a RIL population was developed from a cross between the elite malting cultivar ‘Rasmusson’ (Smith et al., 2010) with moderate FHB susceptibility and the six-rowed landrace PI 383933 with exceptional FHB susceptibility (Huang et al., 2013). The reaction of Rasmusson to FHB is typical of many other six-rowed cultivars and breeding lines in the Minnesota program. The aims of the present study were: (1) to identify possible novel QTL conferring resistance to FHB and DON accumulation in a representative Minnesota barley cultivar (Rasmusson) by means of a cross to a highly susceptible accession (PI 383933); and (2) to characterize the genetic architecture of disease resistance and agromorphological traits in a cross where the exotic parent is early, short and highly susceptible.

## Materials and Methods

### Mapping Population

The mapping population consisted of 93 F_5:7_ RILs developed from a cross between cultivar ‘Rasmusson’ and PI 383933 by single seed descent in St. Paul, MN, United States. In the F_6_ generation, several plants from each line were bulk harvested and the bulks were evaluated for resistance to FHB, DON accumulation and various agronomic traits in the F_5:7_ (2015) and F_5:8_ (2016) generations. Rasmusson is a spring six-rowed malting type cultivar released by the University of Minnesota barley breeding program in 2010 with Chevron in its pedigree (Line record M109^1^) (Blake et al., 2016). It is moderately susceptibility to FHB (Smith et al., 2010) and is typical of cultivars and breeding lines in the University of Minnesota program. PI 383933 is a spring six-rowed landrace originating from Japan (United States National Plant Germplasm System^2^). It is a short-statured, early maturing accession with a dense spike phenotype and is one of the most highly and reliably susceptible accessions known in barley, exhibiting FHB severities of 50–80% in most experiments.

### FHB, DON, and Agronomic Traits Evaluation

Field trials for the RIL population and its parents were conducted in four environments in Minnesota during the 2015 and 2016 growing seasons: two at the University of Minnesota Agricultural Experiment Station (MAES) in St. Paul and two at the University of Minnesota Northwest Research and Outreach Center (NWROC) in Crookston. Entries were seeded as single row plots with a length of 1.8 m in a randomized complete block design (RCBD) with three replications in St. Paul and two replications in Crookston. The following controls were included in the nurseries: Chevron (CI 1111) (a late-maturing, six-rowed landrace accession with partial FHB resistance), Quest (PI 663183) (a midseason, six-rowed malting cultivar with partial FHB resistance) (Smith et al., 2013) and Stander (PI 564743) (a midseason, six-rowed malting cultivar with FHB susceptibility).

Two different inoculation methods were used depending on the location. At St. Paul, each plot was spray-inoculated with a macroconidial suspension (1 × 10^5^ spores/mL, plus 0.02% polyethylene glycol sorbitan monolaurate, Tween^®^ 20, MilliporeSigma, St. Louis, MO, United States) derived from a mixture of 39 *F. graminearum* isolates locally collected 2010–2013 (Huang et al., 2016). The inoculum was applied using a CO_2_-pressured backpack sprayer. The inoculations were made 2–3 days after individual entries had headed (i.e., when 50% of plants in a row had their spikes 50% emerged from the boot) and again 2–4 days after the first inoculation (Nduulu et al., 2007). Mist irrigation was provided for 30 min after each inoculation and then at regular intervals from late afternoon until midnight to promote infection and disease development. The Crookston FHB nursery was inoculated by spreading onto the ground autoclaved maize and barley kernels colonized with a mixture of 19 isolates of *F. graminearum* (5.21 g plot^-1^) (Horsley et al., 2006; Mamo and Steffenson, 2015). Inoculations were made 1 week before the HD of the earliest maturing accessions and then again 1 week later. This timing for inoculation coincided with the development of perithecia and subsequent release of ascospores for infection of newly headed entries. The daily mist irrigation was provided by overhead sprinklers.

For FHB severity assessment, 10 spikes were randomly selected from the top of the canopy within each plot. Then, the number of FHB-infected kernels within each spike and the total number of kernels within each spike (averaged from three spikes) was counted to calculate the percentage of FHB for each spike. In St. Paul, the Rasmusson × PI 383933 population was divided into multiple inoculation groups in 2015 and 2016 based on HD. FHB severity was assessed 16 days after the first spray inoculation for each entry. At Crookston, due to its outstate location, entries were divided into early and late heading groups, with FHB severity being assessed 2 weeks after the recorded HD for each group.

Deoxynivalenol accumulation was determined in harvested grain samples from each plot in Crookston in 2015, and also in St. Paul and Crookston in 2016. For St. Paul 2015, harvested grain from three replications was inadvertently pooled and then assayed for DON concentration. The analytical procedure followed that of Mirocha et al. (1998) and Dong et al. (2006).

Agromorphological traits were assessed for all entries to determine their possible association with FHB severity. These traits included HD, HT, SL, and SD (Steffenson, 2003; Mamo and Steffenson, 2015). HD was recorded as the number of days from planting to the date when 50% of the heads in a plot had emerged halfway or more from the boot. HT was measured as the distance from the base of the plant to the top of the spike excluding awns. SL was measured as the mean distance between the top and bottom spikelets on three randomly selected spikes. SD was calculated as the quotient of the number of rachis nodes divided by the SL.

### Statistical Analysis

Analysis of variance (ANOVA) in individual environments was conducted in R version 3.3.1 (R Core team, 2014) and used individual observations from each replicate for each RIL. Combined ANOVA across four environments for each trait used the mean value of each replicate for each RIL in each environment. The observed trait response, *P*_ijkr__,_ of the genotype (G) *i* in the location (L) *j*, year (Y) k and block (B) *r* was modeled using the formula:

$$P_{ijkr}=m+G_{i}+L_{j}+Y_{k}+B_{r}(L_{j}Y_{k})+{GL}_{ij}+{GY}_{ik}+{LY}_{jk}+{GLY}_{ijk}+e_{ijkr},$$

where m is the grand mean and *e*_ijkr_ is the random error. Fitting the linear regression model and generating the ANOVA table were done using the *lm* and *anova* functions in R, respectively. For estimation of variance components, main effects of G, L and Y were considered to be random, and mean squares (MS) were equated to their expectations (EMS). The harmonic mean of the number of replicates (*r* = 2.4) in each environment was used in estimation of variance components. Broad-sense heritability (*H*) was estimated on progeny-mean basis with the formula

$$H={\sigma_{g}}^{2}/\left[\left({\sigma_{g}}^{2}\right)+\left({\sigma_{gy}}^{2}/y\right)+\left({\sigma_{gl}}^{2}/l\right)+({\sigma_{gyl}}^{2}/yl)+({\sigma_{e}}^{2}/ryl)\right],$$

where σ_g_^2^ is the genotypic variance, σ_gy_^2^ is the genotype by year interaction variance, σ_gl_^2^ is the genotype by location interaction variance, σ_gyl_^2^ is the genotype by year by location interaction variance, σ_e_^2^ is the error variance, *y* is the number of years, *l* is the number of locations, and *r* is the harmonic mean of the number of replicates. For combined analysis across four environments, best linear unbiased prediction (BLUP) (Henderson, 1975) for trait values of each genotype were calculated using the R *lme4* package (Bates et al., 2015) with the assumption that genotype, year and location were random effects. Correlation between traits was analyzed using the R *corrplot* package (Wei and Simko, 2016), where the line mean for each trait in each environment was used as input.

### Single Nucleotide Polymorphism (SNP) Genotyping and Linkage Map Construction

Three-week-old leaf tissue from the parents and a bulk of eight F_5:6_ plants of each RIL were collected for DNA extraction and genotyping analysis. DNA samples were genotyped with the barley iSelect 9K SNP chip at the United States Department of Agriculture-Agricultural Research Service (USDA-ARS) Cereal Crops Research Unit in Fargo, ND, United States. The chip contains a total of 7,842 SNP markers which represent a combination of 2,832 barley Illumina GoldenGate oligonucleotide pool assays (OPA) SNPs (Close et al., 2009) and 5,010 SNPs discovered from next generation sequencing data (Comadran et al., 2012). SNP data were filtered to remove monomorphic markers between Rasmusson and PI 383933 and also markers with greater than 5% missing data. After these filtering steps, 2,628 markers were used in linkage map construction.

Joinmap 4.0 was used to generate a barley genetic map for QTL analysis (Stam, 1993; Van Ooijen, 2006). Markers genetically mapped to the same location (locus pairs with similarity of 1.0) were excluded from further analysis, which reduced the number of markers to 1,394. Linkage grouping was based on tests of independence with LOD (logarithm of odds) scores ranging from 2.0 to 10.0 with a step of 1.0 and using LOD 5.0 as a threshold value. A maximum likelihood (ML) mapping algorithm was used because it is more efficient in mapping high density markers compared to a regression mapping algorithm (Jansen et al., 2001). Default ML mapping parameters were applied. Linkage groups and map order were compared with the barley consensus map (Muñoz-Amatriaín et al., 2014) to assign the barley chromosome designation of 1H to 7H.

For haplotype analysis, Chevron and Stander were genotyped using the same platform as that used for the mapping population. Haplotypes consisted of markers selected from the confidence interval of a QTL region and that differentiated genotypes.

### QTL Analysis

Windows QTL Cartographer V2.5 was used for QTL identification in a single environment (Wang et al., 2012). The mean trait value of each RIL within an environment was used as input for CIM analysis (Zeng, 1994). The QTL mapping based on individual environment strategy facilitated the detection of QTL by environment interaction. The threshold level for determining QTL was generated via 1,000 permutations for each trait to achieve the experimental-wise error rate of less than 0.05. The walk speed for genome scan interval was set to 1 cM. Other CIM control parameters used were as suggested by the manual: Model 6-standard model, Control marker numbers-5, window size-5 cM and regression method 3-forward and backward method. The presence of QTL was declared if the peak LOD score exceeded the threshold level. QTL were automatically located by the software using 5 cM as the minimum distance between QTL and 1.0 as the minimum LOD from top to valley. The summary QTL information file (EQTL file) was created, and the results were used to prepare QTL tables. Visualization of QTL locations on barley chromosomes was performed using MapChart 2.32 (Voorrips, 2002). Assignment of QTL regions to chromosome bins were based on the Oregon Wolfe barley mapping population (Szücs et al., 2009)

## Results

### Phenotypic and Genetic Variability of the Mapping Population

**FIGURE 1:**
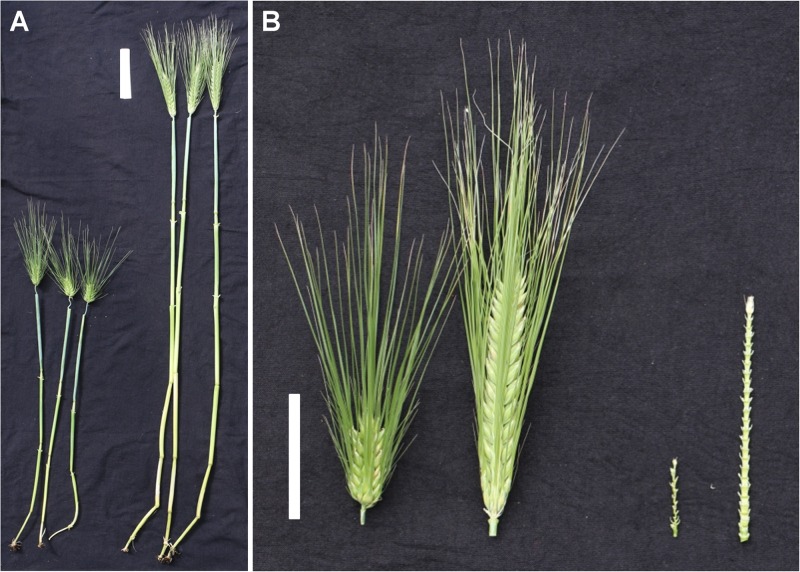
Differences in plant morphology between the mapping parents PI383933 and Rasmusson. **(A)** Comparison of plant height 2 weeks after heading. White bar equals 10 cm; and **(B)** Morphology of spikes (left) and rachises (right) reflecting differences in spike density. White bar equals 5 cm. For each comparison, PI383933 is on the left and Rasmusson on the right.

**FIGURE 2:**
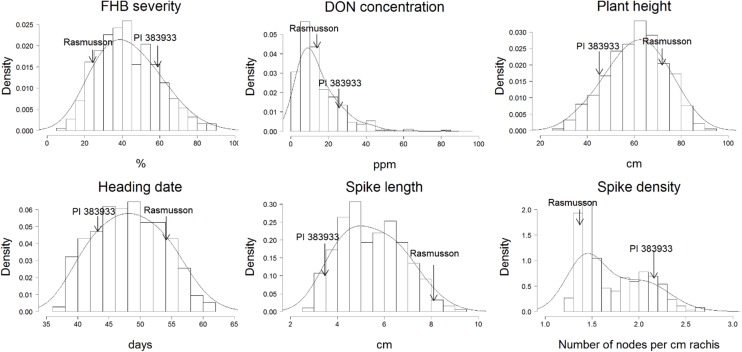
Frequency distribution of trait observations in the Rasmusson × PI383933 recombinant inbred line (RIL) population. Values represented mean of each RIL within individual environment. Parental values for each trait were indicated by arrows. *X*-axis represents the unit of measurement. *Y*-axis represents the probability density estimation of the frequency of lines.

The mean values and ranges for the traits examined in separate environments are shown in **Table 1**. In all four environments, the controls performed as expected, validating that the experimental design and disease pressure could differentiate the responses of genotypes. The mean FHB severity was 8.0%, 16.8%, and 29.7% for Chevron, Quest, and Stander, respectively. Rasmusson exhibited lower FHB severity and DON accumulation than the highly susceptible parent PI 383933. FHB severity ranged from 12.9 to 31.0% for Rasmusson and from 46.2 to 73.8% for PI 383933. DON concentration in harvested grain ranged from 9.2 to 22.4 ppm for Rasmusson and from 15.3 to 46.5 ppm for PI 383933. In addition, Rasmusson had a later HD (by 10–12 days), greater HT (by 15–30 cm), longer SL (by 4–5 cm) and lower SD (by 37%) than PI 383933 (**Table 1** and **Figure 1**). Significant variation among the RIL families was observed for each trait except DON accumulation at St. Paul 2015 due to the accidental pooling of the samples. The population means of each trait were close to the mid-parent values in all environments, indicating that all traits were controlled by QTL with additive effects. Frequency distributions of trait data for FHB, DON, HD, HT, and SL showed patterns close to a normal curve (**Figure 2**). The only exception was with SD, which displayed a bimodal distribution, suggesting that it is controlled by one major QTL in this population. Bi-directional transgressive segregants were identified for each trait in one or more environments.

**Table 1:** Parental means, population means and range, and ANOVA tests of line means in the Rasmusson/PI383933 recombinant inbred line (RIL) population for Fusarium head blight (FHB), deoxynivalenol accumulation (DON), heading date (HD), plant height (HT), spike length (SL), and spike density (SD) in St. Paul (StP) and Crookston (CR).

Trait	Environment	Rasmusson	PI383933	Population	Population range	Significance^†^
FHB	StP 2015	12.9	62.4	45.5	12.4–88.5	^∗∗∗^
(% severity)	CR 2015	27.7	46.2	46.1	19.1–79.9	^∗∗∗^
	StP 2016	25.6	73.8	39.9	8.5–76.9	^∗∗∗^
	CR 2016	31.0	53.1	41.1	13.4–85.5	^∗^
	Mean	24.3	58.9	43.1	8.5–88.5	
DON (ppm)	StP 2015	9.9	46.5	20.5	7.4–52.1	na
	CR 2015	14.2	16.1	14.7	3.3–62.8	^∗∗∗^
	StP 2016	9.2	15.3	8.9	1.7–39.8	^∗∗∗^
	CR 2016	22.4	24.7	13.9	1.1–81.2	^∗^
	Mean	13.9	25.7	14.5	1.1–81.2	
HD (d)	StP 2015	56.7	44.7	50.5	42.7–62.0	^∗∗∗^
	CR 2015	58.0	48.0	52.6	46.0–62.0	^∗∗∗^
	StP 2016	51.3	40.0	44.7	37.3–57.0	^∗∗∗^
	CR 2016	50.5	40.0	46.1	40.0–57.5	^∗∗∗^
	Mean	54.1	43.2	48.5	37.3–62.0	
HT (cm)	StP 2015	82.0	52.3	68.8	43.3–87.0	^∗∗∗^
	CR 2015	81.5	47.5	65.1	43.5–91.5	^∗∗∗^
	StP 2016	59.0	44.7	58.3	38.0–73.3	^∗∗∗^
	CR 2016	64.5	36.0	51.6	26.5–75.5	^∗∗∗^
	Mean	71.8	45.1	60.9	26.5–91.5	
SL (cm)	StP 2015	7.8	3.2	5.2	3.1–7.5	^∗∗∗^
	CR 2015	8.6	3.8	5.5	3.2–8.3	^∗∗∗^
	StP 2016	7.6	3.5	5.6	3.2–8.6	^∗∗∗^
	CR 2016	8.4	3.4	5.5	2.9–9.2	^∗∗∗^
	Mean	8.1	3.5	5.5	2.9–9.2	
SD (number of nodes per cm rachis)	StP 2015	1.33	2.03	1.66	1.21–2.36	^∗∗∗^
	CR 2015	1.36	2.13	1.71	1.25–2.66	^∗∗∗^
	StP 2016	1.42	2.14	1.71	1.26–2.68	^∗∗∗^
	CR 2016	1.37	2.35	1.71	1.25–2.61	^∗∗∗^
	Mean	1.37	2.16	1.70	1.21–2.68	


**^†^*ANOVA test for significant variability among RIL families for the indicated trait and environment. na, not available. Test result unavailable for DON StP 2015 due to inadvertent pooling of replicates. ^∗^Significant at *p* < *0*.05 level, ^∗∗∗^significant at *p* < 0.001 level.*

Analysis of variance across environments revealed significant differences among genotypes (G), years (Y), and locations (L). In addition, there were significant genotype-by-location (G × L) and genotype-by-year (G × Y) interactions for all traits (**Table 2**). The higher order interactions of G × L × Y were significant for all traits except for SD. Broad-sense heritability estimates the proportion of total phenotypic variance that is due to genetic variance, taking into account additive, dominant and epistatic genetic variances. The heritability estimates within the mapping population were low for FHB at 0.30, moderate for DON at 0.65 and high for HD, HT, SL and SD at 0.96, 0.88, 0.96, and 0.97, respectively. These estimates were comparable with what were reported in previous FHB mapping studies (Mesfin et al., 2003; Massman et al., 2011).

**Table 2:** ANOVA analyses across environments and heritability estimation on a progeny mean basis in the Rasmusson/PI 383933 recombinant inbred line (RIL) population for Fusarium head blight (FHB), deoxynivalenol accumulation (DON), heading date (HD), plant height (HT), spike length (SL), and spike density (SD).

Trait	Variance components						*H*^‡^
		
	MS_g_^†^	MS_l_	MS_y_	MS_g:l_	MS_g:y_	MS_g:y:l_	
FHB	1227.2^∗∗∗^	110.9	7008.5^∗∗∗^	543.0^∗∗∗^	533.4^∗∗∗^	223.2^∗^	0.30
DON	500.9^∗∗∗^	159.4^∗^	5936.1^∗∗∗^	144.4^∗∗∗^	63.9^∗∗∗^	31.3	0.65
HD	187.6^∗∗∗^	669.5^∗∗∗^	8604.4^∗∗∗^	4.9^∗∗∗^	5.5^∗∗∗^	2.9^∗∗∗^	0.96
HT	880.8^∗∗∗^	6061.9^∗∗∗^	31540.7^∗∗∗^	86.0^∗∗∗^	39.1^∗∗∗^	22.1^∗^	0.88
SL	15.9^∗∗∗^	1.8^∗∗^	18.1^∗∗∗^	0.3^∗∗^	0.6^∗∗∗^	0.3^∗∗^	0.96
SD	1.07^∗∗∗^	0.13^∗^	0.19^∗∗^	0.03^∗^	0.03^∗∗^	0.02	0.97


*^†^Mean square, g-genotype, l-location, y-year. ^∗^Significant at *p* < *0*.05 level, ^∗∗^significant at *p* < 0.01 level, ^∗∗∗^significant at *p* < 0.001 level. ^‡^Broad-sense heritability based on progeny means across four environments.*

### Correlation of FHB, DON, and Agromorphological Traits

**FIGURE 3:**
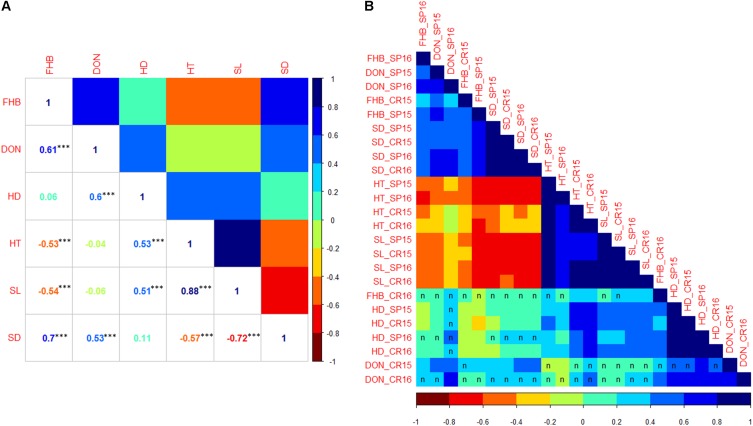
**(A)** Correlation coefficients among traits based on BLUP values across all four environments. ^∗∗∗^Significant at *p* < 0.001. **(B)** Heat map of the correlation matrix among traits based on mean values of each RIL within individual environment. Matrix was ordered by hierarchical clustering. Correlation between environments was significant at *p* < 0.05 level unless indicated by letter ‘n.’ FHB, Fusarium head blight; DON, deoxynivalenol accumulation; HD, heading date; HT, plant height; SL, spike length; SD, spike density.

To obtain an overview of the correlation among all traits, BLUP values were calculated across all four environments for each trait and displayed on a scatterplot matrix (**Supplementary Figure S1**). The correlation coefficients based on BLUPs suggested that FHB severity was positively correlated with those for DON accumulation (0.61) and SD (0.70), negatively correlated with those for HT (-0.53) and SL (-0.54) and showed no obvious pattern of correlation with HD (**Figure 3A**). DON accumulation showed positive correlation with HD (0.60) and SD (0.53) and no correlation with HT and SL. HT showed positive correlation with SL (0.88) and negative correlation with SD (-0.57). Among individual environment comparisons, FHB severity showed significant and positive correlations in three out of four environments (**Figure 3B**, for correlation coefficients, see **Supplementary Table S1**). At Crookston 2016, FHB severity was not significantly correlated with FHB severity from the other environments. DON accumulation was positively and significantly correlated in five out of six comparisons. Significant and positive correlations among environments were observed for each of the agromorphological traits, i.e., HD, HT, SL, and SD.

Among the observed traits, FHB severity showed positive and significant correlations with SD in 12 out of 16 comparisons. Significant and negative correlations between FHB severity and HT and SL were observed in three out of four environments. The FHB severity at Crookston 2016 showed positive and significant correlations with HT and SL in three of the four cases. FHB severity was not significantly correlated with HD, with the exception of Crookston 2016 where it showed significant and positive correlations with HD. DON accumulation was positively correlated with FHB severity in all four environments. Among agromorphological traits, SD showed significant and negative correlations with HT and SL, and both positive and negative correlations with HD. HD, HT, and SL were significantly and positively correlated.

### Genetic Linkage Map

A total of 1,394 polymorphic SNP markers were used to construct the genetic map, which comprised seven linkage groups totaling a genetic distance of 1,328 cM (**Table 3** and **Supplementary Figure S2**). The assignment and order of markers on the barley chromosomes are consistent with the iSelect 9K consensus genetic map (Muñoz-Amatriaín et al., 2014). The average marker interval for the map is 1.3 cM. There were from one to three large gaps (>9 cM) in each of the barley chromosomes with the exception of chromosome 3H (**Table 3**). Overall, the genome is evenly covered by markers.

**Table 3:** Distribution and density of SNP markers in the Rasmusson × PI383933 recombinant inbred line (RIL) population.

Chromosome	Total SNPs	Unique loci^†^	Length (cM)	Density	Gaps (cM/locus)
					(cM intervals)^‡^
1H	132	106	181.8	1.7	94.7–111.4, 118.7–136.6, 139.7–155.4
2H	232	176	217.5	1.2	147.2–157.9
3H	217	154	191.6	1.2	
4H	179	134	185.6	1.4	79.9–92.8, 92.8–102.8
5H	254	188	218.9	1.2	64.9–75.2, 175.6–185.0
6H	187	134	147.6	1.1	11.8–22.2
7H	193	149	185.2	1.2	118.6–127.8, 127.8–138.8
Mean	199	149	189.8	1.3	
Total	1394	1041	1328.3		


*^†^Number of loci that segregate independently. ^‡^Large regions (>9 cM) with no marker coverage.*

The mean homozygosity was 94.51% in the mapping population, which is close to but significantly higher (χ^2^ = 129.5, *df* = 1, *p* < 0.001) than the expected homozygosity of 93.75% in an F_5_-derived RIL population. The proportion of the Rasmusson allele in the population is 48.7%. A goodness of fit test suggested a significant deviation of the observed allele frequency from the expected 1:1 ratio (χ^2^ = 77.4, *df* = 1, *p* < 0.001). Of the 1,394 markers, 171 showed significant segregation distortion (*p* < 0.05). Rasmusson alleles were over-represented at 134 loci, while PI 383933 alleles were over-represented at 37 loci. Regions on the long arms of chromosome 4H and 7H were biased toward PI 383933 alleles, whereas the proximal end of the 5H long arm and short arms of 6H and 7H were skewed toward Rasmusson alleles. The segregation distortion observed might be due to the small population size and/or genetic factors affecting gametic or zygotic viability.

### QTL Analysis

**FIGURE 4:**
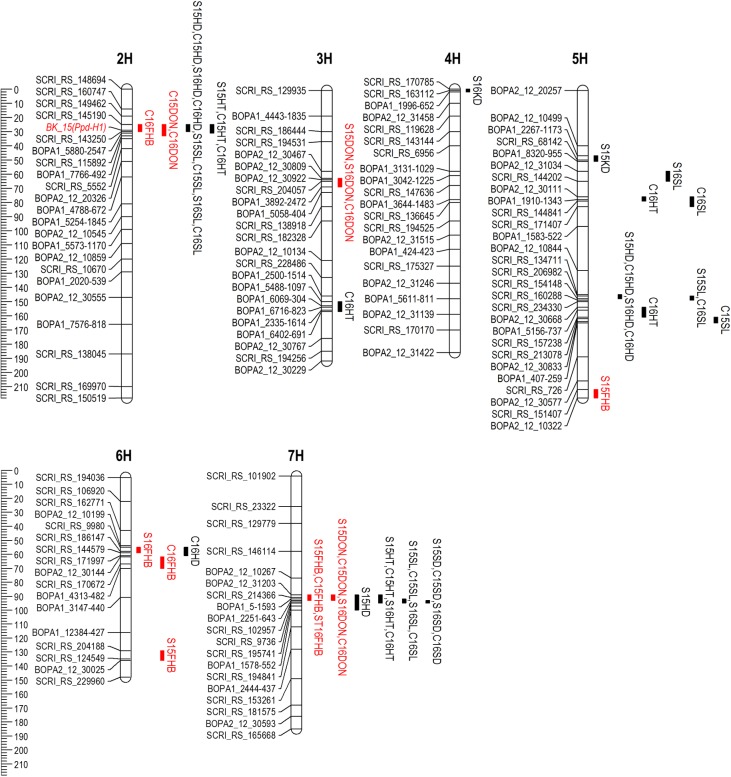
Chromosomal locations of QTL detected for FHB resistance (red), DON accumulation (red) and agro-morphological traits in the Rasmusson × PI383933 recombinant inbred line (RIL) population in four environments. QTL intervals represent one LOD score drop. QTL name is denoted by location (S, St. Paul; C, Crookston), year (2015 or 2016) and trait. FHB, Fusarium head blight; HD, heading date; HT, plant height; SL, spike length; SD, spike density.

A total of six QTL were identified for FHB severity on 2H, 5H, 6H, and 7H (**Table 4** and **Figure 4**). QTL positions were assigned to bin locations based on the Oregon Wolfe Barley mapping population (Szücs et al., 2009). Rasmusson contributed the resistance alleles for QTL on chromosomes 6H and 7H, whereas PI 383933 contributed the resistance alleles for QTL on chromosomes 2H, 5H, and 6H. The QTL on chromosome 7H had the largest effect, was significant in three out of four environments, and is located near the centromere of chromosome 7H between markers BOPA2_12_31203 and BOPA1_2251-643. The phenotypic variance explained by this QTL ranged from 18.6% at Crookston 2015 to 33.6% at St. Paul 2015. The Rasmusson allele at this QTL contributed to a 5.8–10.5% reduction in FHB severity. The 7H FHB QTL was coincident with QTL for DON accumulation, HT, SL and SD. The other FHB QTL identified had relatively minor effects on the phenotype and were only detected in a single environment. The alleles for two of these minor effect FHB QTL were contributed by Rasmusson and resided in linked regions of 6H (bin 6-7) at St. Paul 2016 and Crookston 2016. The distance between the peaks of these two QTL regions was only 8.9 cM, suggesting that a single QTL was likely present in the 6H bin 6-7 region. At St. Paul 2015, two FHB QTL were identified near the telomeres of 5HL and 6HL. The PI 383933 alleles contributed the resistance at these QTL with *R*^2^ values of ranging from 5.5 to 6.2%. At Crookston 2016, one FHB QTL was detected in the 2H bin 3 region with PI 383933 providing the resistance allele. This QTL was coincident with QTL for DON accumulation, HD, HT, and SL.

**Table 4:** Quantitative trait loci associated with FHB resistance (FHB), DON accumulation (DON), plant height (HT), heading date (HD), spike length (SL), and spike density (SD) in the Rasmusson × PI383933 recombinant inbred line (RIL) population.

Trait	Chr. (Bin)^a^	Marker position (cM) (confidence interval)^b,c^	St. Paul 2015			Crookston 2015			Saint Paul 2016			Crookston 2016		
						
			LOD	*R*^2^ (%)^d^	Effect^e^	LOD	*R*^2^ (%)	Effect	LOD	*R*^2^ (%)	Effect	LOD	*R*^2^ (%)	Effect
FHB	2H(3)	28.7 (S_145190–S_143250)										8.56	29.0	8.51
	5H(15)	212.7 (S_151407–B2_12_10322)	3.43	6.2	5.05									
	6H(6)	58.0 (S_9980–S_144579)							3.92	9.8	-5.60			
	6H(6-7)	66.9 (B2_12_30144–B1_4313-482)										3.50	9.7	-5.23
	6H(14)	133.1 (S_204188–B2_12_30025)	3.16	5.5	4.37									
	7H(7)	92.0 (B2_12_31203–B1_2251-643)	12.43	33.6	-10.48	5.93	18.6	-5.82	7.56	22.6	-7.61			
DON	2H(3)	30.8 (S_145190–S_115892)				4.41	13.0	3.80				6.21	16.8	5.55
	3H(4)	65.5 (B2_12_20574–B1_3892-2472)	4.64	9.8	-3.67				6.61	15.2	-2.72	3.70	9.1	-4.15
	7H(7)	92.0 (B2_12_31203–B1_2251-643)	10.12	25.5	-5.50	5.40	16.8	-4.31	11.75	35.6	-4.17	3.78	9.8	-4.22
HD	2H(3)	28.7 (S_145190–S_143250)	27.80	56.5	3.37	31.07	53.8	2.97	24.76	53.4	3.76	30.17	64.5	3.53
	5H(11)	148.3 (S_134711–S_154148)	10.54	13.2	1.67	7.00	6.3	1.01	7.26	9.0	1.52	5.71	6.7	1.14
	6H(6)	55.2 (S_9980–S_171997)										3.33	3.7	-0.90
	7H(7)	97.2 (B2_12_31203–B1_1578-552)	3.59	3.4	-0.82									
HT	2H(3)	30.1 (S_145190–B1_5880-2547)	8.52	11.7	3.42	20.53	34.6	6.87				22.56	42.1	7.75
	3H(13)	153.0 (B1_5488-1097–B1_6402-691)										3.66	3.2	-2.16
	5H(6)	78.7 (B2_12_30111–S_144841)										7.02	6.4	3.05
	5H(11)	158.4 (B2_12_30668–S_157238)										7.57	7.2	3.20
	7H(7)	93.0 (B2_12_31203–S_9736)	11.77	22.1	4.73	3.47	10.7	3.95	9.63	22.9	4.05	5.58	5.8	2.91
SL	2H(3)	29.1 (S_145190–S_143250)	14.32	17.2	0.48	15.49	19.6	0.58	14.93	24.6	0.67	16.61	27.3	0.80
	5H(6)	68.9 (B2_12_31034–S_144202)							5.30	5.5	0.39			
	5H(6)	78.7 (B2_12_30111–S_171407)										4.08	3.9	0.31
	5H(11)	148.3 (S_206982–S_160288)	5.53	5.5	0.27							7.69	8.3	0.44
	5H(11)	162.2 (S_197532–B1_407-259)				3.91	3.7	0.25						
	7H(7)	94.1 (B1_5-1593–B1_3010-1391)	20.69	39.7	0.72	18.88	35.3	0.78	11.87	19.3	0.61	11.75	18.3	0.66
SD	4H(1)	0.6 (S_170785-B1_1996-652)							5.89	5.0	-0.08			
	5H(4)	49.1 (B1_2267-1173–B1_8320-955)	3.74	3.0	-0.054									
	7H(7)	94.1 (B1_2251–643-S_9736)	25.68	58.6	-0.24	28.90	70.2	-0.31	22.39	48.3	-0.26	25.20	59.7	-0.27


*^*a*^Bin location of QTL was inferred from Oregon Wolfe barley mapping population (Szücs et al., 2009). ^*b*^CI represents one LOD score drop on either side of the QTL peak. ^*c*^Abbreviations for markers, S = SCRI_RS, B1 = BOPA1, B2 = BOPA2. ^*d*^Percentage of phenotypic variance explained by QTL. ^*e*^Effect of Rasmusson allele, minus sign indicates reduction in the trait value.*

Three QTL were detected for DON accumulation. Rasmusson contributed the alleles for lower DON concentration on the 3H and 7H QTL, whereas PI 383933 contributed the resistant allele at the 2H QTL. The DON QTL near the centromere of 7H was detected in all four environments and explained between 9.8 and 35.6% of the phenotypic variation. The QTL in 3H bin 4 region was found in St. Paul 2015 and 2016 and Crookston 2016, and explained between 9.1 and 15.2% of the phenotypic variation. The QTL in 2H bin 3 region was found in Crookston 2015 and 2016, explained between 13.0 and 16.8% of the phenotypic variation and coincided with QTL for FHB, HD, HT, and SL.

Four QTL were discovered for HD on 2H, 5H, 6H, and 7H. The QTL on 2H and 5H were detected in all four environments. The marker (BK_15, **Figure 4**) closest to the QTL peak in 2H bin 3 is located in the *Ppd-H1* gene which regulates photoperiod response and flowering time in barley (Turner et al., 2005). This gene explained 53.4–64.5% of the phenotypic variation for HD with the Rasmusson allele delaying heading by 3.4 days on average. The QTL on 5H explained 6.3–13.2% of phenotypic variance with the Rasmusson allele delaying heading by a mean value of 1.3 days. One QTL in 6H bin 6 and another in 7H bin 7 were associated with HD. In both cases, the Rasmusson allele contributed to early heading by approximately 1 day.

Five QTL for HT were detected on 2H, 3H, 5H, and 7H. The Rasmusson alleles contributed to increased height for all QTL except for the one on 3H. The 2H HT QTL region was coincident with the HD QTL and explained 11.7–42.1% of phenotypic variance. The 7H QTL explained 5.8–22.9% of the variance in height. The three HT QTL in 5H bin 6, 5H bin 11 and 3H bin 13 were detected only at Crookston 2016. The two HT QTL on 5H were coincident with QTL for SL.

Six QTL were found to control SL with the Rasmusson alleles contributing to increased SL in all cases. The QTL on 2H and 7H were detected in all four environments and were coincident with QTL for HD (2H) and HT (2H and 7H). In 2015, the 7H QTL explained the largest phenotypic variance across both locations (39.7% and 35.3%). In contrast, the 2H QTL explained the most phenotypic variance across the two locations in 2016 (24.6% and 27.3%), indicating a QTL × environment interaction. In the 5H bin 6 region, a QTL was detected at St. Paul 2016 and another was detected at Crookston 2016. The distance between the two QTL peaks was less than 10 cM, suggesting that they were likely one QTL. In the 5H bin 11 region, one QTL was detected at St. Paul 2015 and Crookston 2016, and the other QTL was detected at Crookston 2015. The proximity of the two QTL regions indicated that they might represent one QTL detected in different environments.

Three QTL were detected for SD on 4H, 5H, and 7H. The Rasmusson alleles were responsible for a reduced SD at all three QTL and contributed to a lax-spike phenotype. The 7H QTL was found in all environments and explained from 48.3 to 70.2% of phenotypic variance and was coincident with QTL for HT and SL. Two additional SD QTL were detected in just a single environment: one in 4H bin 1 and the other in 5H bin 4. These QTL explained a much smaller phenotypic variance than the 7H QTL and were detected at St. Paul 2015 and St. Paul 2016, respectively. These two QTL were not coincident with QTL for other traits.

To determine whether the 3H DON QTL was derived from Chevron, marker haplotypes at this QTL (DON3H.66) were compared among Chevron, Rasmusson, PI383933, and Stander (**Table 5**). In this region, Rasmusson and Stander had the same haplotype but were different from that of Chevron, and the PI 383933 accession was different from all three. This result suggests that the 3H DON QTL represents native resistance in the elite germplasm in the University of Minnesota breeding program.

**Table 5:** Marker haplotypes of Chevron, Rasmusson, PI 383933, and Stander in the chromosome 3H DON QTL region.

Marker	Position (cM)	QTL region	Chevron	Rasmusson	PI 383933	Stander
BOPA2_12_20574	62.2	DON3H.66	BB	AA	BB	AA
BOPA2_12_30583	62.8		AA	AA	BB	AA
BOPA2_12_30922	63.9		BB	BB	AA	BB
BOPA2_12_30923	63.9		AA	AA	BB	AA
SCRI_RS_204057	64.5		BB	AA	BB	AA
BOPA2_12_11387	65.4		BB	BB	AA	BB
BOPA1_3892-2472	69.1		AA	BB	AA	BB


## Discussion

### Detection of Resistance QTL to FHB and DON in an Elite Barley Cultivar

Most of the previous mapping studies for FHB resistance utilized exotic sources of resistance crossed to susceptible elite cultivars or breeding lines from the Upper Midwestern United States, the region that was historically most severely impacted by the disease. Since the FHB-resistant landraces Chevron and Peatland (Åberg and Wiebe, 1946) were used as founder parents in the University of Minnesota and North Dakota State University barley breeding programs, it is possible that some minor native resistance QTL remained undetected in the elite germplasm.

In this study, a population was derived from crossing a moderately susceptible six-rowed spring malting barley cultivar developed by the University of Minnesota (‘Rasmusson’) with the most susceptible barley accession (PI 383933) identified from extensive evaluations of *Hordeum* germplasm (Huang et al., 2013). PI 383933 is unadapted to the United States Upper Midwestern region and has unfavorable agronomical traits such as early heading, short stature and dense spikes. However, it is highly susceptible to FHB even when compared with elite cultivars. The rationale of using PI 383933 was to increase the genetic variability between the mapping parents and the chance of identifying FHB/DON QTL that are present in the elite germplasm.

One caveat of using a relatively small population size (93 RILs) was that the genetic effects of QTL might be overestimated due to the Beavis effect (Beavis, 1994, 1998; Xu, 2003). A large population size is required to achieve a reasonable estimation (for example, 500 progeny) (Xu, 2003), which is not practical with the FHB QTL mapping effort that is quite labor-intensive. Previous studies have used population sizes comparable to this study and successfully identified FHB/DON QTL (de la Peña et al., 1999; Dahleen et al., 2003; Mesfin et al., 2003). Thus, the use of 93 RILs is sufficient for the purpose of discovering disease QTL in this specific cross.

Rasmusson is closely related to several moderately susceptible/susceptible parents (Stander and breeding line M69) used in previous bi-parental QTL mapping studies (de la Peña et al., 1999; Ma et al., 2000; Mesfin et al., 2003) and therefore may carry minor but not major FHB QTL identified in exotic resistance sources. Indeed, we did not detect any resistance QTL in three of the common regions associated with FHB resistance and DON accumulation, namely the 2H bin 8, 2H bin 10 and 2H bin 13 regions (Massman et al., 2011). Six FHB QTL and three DON QTL were identified in the Rasmusson/PI 383933 mapping population used in this study. The largest effect QTL identified for FHB severity and DON accumulation was in the chromosome 7H bin 7 region and coincided with QTL for SD, SL, and HT (**Table 4**). Two closely linked FHB resistance QTL were detected on chromosome 6H bin 6-7 and they are considered to represent the same QTL. This 6H QTL also coincided with previously identified QTL in this region from Chevron, Fredrickson, and breeding lines from the Upper Midwest region of the United States (Mesfin et al., 2003; Canci et al., 2004; Massman et al., 2011). The detection of the 6H FHB QTL from the Rasmusson × PI383933 mapping population suggested that an allelic series for the 6H bin 6-7 FHB QTL might exist among late unadapted moderately resistant landrace Chevron, elite moderately susceptible cultivar Rasmusson and early highly susceptible landrace PI383933, with the allelic effect for FHB resistance ranking in decreasing order from Chevron to Rasmusson to PI 383933.

A minor effect QTL for DON accumulation was detected in 3H bin 4 region in the Rasmusson/PI 383933 population and the Rasmusson allele reduced DON accumulation. Smith et al. (2004) identified a DON QTL on 3H between markers Bmag0122 and Bmac0067, which is close to the bin 4 region. This QTL was detected in a RIL population derived from Stander, a Minnesota cultivar closely related to Rasmusson, and Fredrickson, a Japanese landrace with FHB resistance. The Stander allele contributed to lower DON in this case. Comparison of marker haplotypes in the 3H DON QTL region revealed that Rasmusson and Stander had the same haplotype, and it differed from Chevron (**Table 5**). This result suggested that the 3H DON QTL represents native resistance in the elite germplasm. In a GWAS study of advanced barley breeding lines from four breeding programs in the Upper Midwest region, Massman et al. (2011) detected a QTL for reduced DON concentration in the same region. The effect of this DON QTL was later validated using NIL pairs by Navara and Smith (2014). In the telomeric regions of 5HL and 6HL, alleles from PI 383933 were associated with reduced FHB severity in one environment and were not coincident with any agromorphological traits. Prior to be utilized in marker assisted selection for FHB and DON resistance, these QTL need to be validated with independent genetic materials. The detection of favorable alleles from a highly susceptible genotype highlights the complex genetics of FHB resistance in barley, but also the potential for mining new resistance alleles from susceptible sources. Of note is that previous FHB mapping studies have also identified resistant alleles from susceptible parents (Zhu et al., 1999; Ma et al., 2000; Mesfin et al., 2003).

### Agromorphological Factors Affecting FHB Susceptibility in PI 383933

Previous mapping studies have shown that FHB and DON resistance QTL often coincided with QTL underlying agronomic and morphological traits (de la Peña et al., 1999; Zhu et al., 1999; Ma et al., 2000; Dahleen et al., 2003; Mesfin et al., 2003; Canci et al., 2004; Hori et al., 2005; Horsley et al., 2006; Sato et al., 2008; Mamo and Steffenson, 2015). Plants with lower FHB severities usually have one or more of the following traits: late heading, increased height and two-rowed spike morphology (Parry et al., 1995; Steffenson et al., 1996; Rudd et al., 2001). The coincidence of QTL for correlated traits may be due to the close linkage of genes controlling the traits or the pleiotropic effects of agromorphological traits on FHB severity. Late heading and tall-statured barley plants may serve as an escape mechanism from *F. graminearum* infection. Late-maturing plants may head during a time in the summer when the infection period is less suitable for infection, and tall plants avoid higher concentrations of inoculum near the soil surface and also longer and heavier dew periods that favor *Fusarium* infection. Two-rowed spikes may present a less favorable micro-environment for disease progression because they have a more open inflorescence structure than that of six-rowed spikes.

In this study, two HD QTL (2H and 5H) were consistently detected in all four environments and the Rasmusson alleles contributed to late heading. The 2H bin 3 HD QTL was coincident with one FHB QTL (Crookston 2016) and two DON QTL (Crookston 2015 and Crookston 2016). In all three cases, the Rasmusson allele conditioned higher FHB severity or DON accumulation. This is in contrast to the findings of previous FHB mapping studies utilizing elite lines crossed with late unadapted germplasm where late HD was associated with lower disease in the 2H bin 8 region (de la Peña et al., 1999; Ma et al., 2000; Canci et al., 2004; Horsley et al., 2006). Interestingly, the 5H HD QTL was not associated with any disease QTL in the study. These results suggested that the relationships between HD QTL and disease QTL (coupling, repulsion or no association) depended on the genotypes of mapping parents, the genetic architecture controlling the two traits, and the environments in which the mapping population was evaluated. The underlying mechanism of coincident HD and disease QTL could be due to tight linkage or pleiotropy. In the 2H bin 8 region, studies have provided evidence that the repulsion relationship between HD and FHB/DON QTL is due to close linkage rather than pleiotropy (Nduulu et al., 2007; Massman et al., 2011). However, the coupling relationship between HD and FHB/DON QTL in this study is likely due to pleiotropic effect of HD QTL for the following two reasons. First, the marker that underlies the 2H bin 3 QTL peak (BK_15) was in the *Ppd-H1* gene, which is known to regulate barley flowering time through photoperiod response (Turner et al., 2005). Second, the association between HD and FHB/DON was detected only in Crookston but not in St. Paul which argued against a resistance locus tightly linked to the *Ppd-H1* gene. The major difference between the St. Paul and Crookston nurseries was the inoculation method. At the St. Paul location, the possible confounding effect of HD was partially mitigated by spray-inoculating lines based on their HD. At the Crookston location, the grain spawn inoculation method was used, which produced ascospore inoculum throughout the heading period of the population. FHB severity in Crookston was scored based on two HD groupings: an early heading group similar to PI 383933 and a normal heading group similar to Rasmusson. The positive association of HD and disease in Crookston was unexpected. However, such positive correlation has been reported previously and the authors postulated that weather conditions after different inoculations might be the reason (Mesfin et al., 2003). For the 2-week periods prior to disease phenotyping of the two heading groups in Crookston 2016, the average daily minimum temperature and the average daily minimum relative humidity (RH) were both higher (by 2.4°C in temperature and 11.3% in RH, respectively) for the normal heading group than for the early heading group (University of Minnesota Northwest Research and Outreach Center, 2016). These weather conditions during the critical heading stage for the normal maturity group (Rasmusson-like) were more favorable for pathogen infection and progression.

The largest effect QTL for resistance to FHB and DON accumulation identified in the Rasmusson/PI 383933 population coincided with QTL for HT, SL, and SD in the centromeric region of 7H. This QTL explained a large proportion of the phenotypic variance for SD (48.3–70.2%), SL (18.3–39.7%), and HT (5.8–22.9%). Lower disease and DON were associated with taller plants with less dense spikes. Previous studies have shown that both dense spike 1 (*dsp1*) and dense spike-ar (*dsp.ar*) map to the centromeric region of chromosome 7H and may represent an allelic series of the same locus (Taketa et al., 2011; Shahinnia et al., 2012). The SD QTL detected on 7H in this study is coincident with the *dsp1/dsp.ar* locus. Takeda and Heta (1989) identified FHB resistant barley lines with both dense spikes and lax spikes. Steffenson et al. (1996) reported that dense spike NILs had higher FHB severity than lax spike NILs, which is consistent with our study, however, the difference was insignificant. Yoshida et al. (2005) compared a NIL pair with a normal and dense spike morphology and found no significant difference within the pair. Zhu et al. (1999) identified coincidental QTL for Type-I resistance and inflorescence density on 3HS and FHB resistance was associated with lax spike. With regard to FHB and HT, it has been shown that FHB severity exhibited a negative correlation with HT (Buerstmayr et al., 2004; Choo et al., 2004). Previous mapping studies have revealed coincident QTL for FHB and HT (de la Peña et al., 1999; Zhu et al., 1999; Ma et al., 2000; Mesfin et al., 2003; Horsley et al., 2006). However, only one study (de la Peña et al., 1999) identified the association of FHB severity and DON accumulation with HT in the centromeric region of chromosome 7H in one environment. In that study, the Chevron allele contributed to reduced DON accumulation but increased HT and FHB severity. In this study, the coincidental QTL for FHB, DON accumulation, HT, SL, and SD in the centromeric region of 7H in most of the environments suggests that high FHB susceptibility in PI383933 could be due to the pleiotropic effects of reduced HT and/or increased SD, although the issue of tight linkage versus pleiotropy could not be unambiguously determined due to the limited mapping resolution. Plant height is inversely correlated with SD in this population. Plants with short stature and dense spikes provide favorable conditions for fungal infection and development. Airborne ascospores are the most important propagule for initiating FHB epidemics on wheat (Fernando et al., 1997) and barley. There is a strong gradient of ascospore concentration from the soil surface to upper plant canopy (Paul et al., 2004). Moreover, the dews are heavier and longer on plant surfaces closer to the soil. Thus, short plants tend to have higher infection levels on the heads due to their proximity to higher spore concentrations at the soil level and a more humid micro-environment compared to plants of tall stature. In addition, densely arranged spikelets on the rachis may also facilitate fungal spread within spikes. Our results support a major tenant that plant architecture and inflorescence traits must be taken into full consideration when breeding barley for FHB resistance.

## Data Availability

The raw data supporting the conclusions of this manuscript can be found at T3/Barley^3^.

## Author Contributions

YH, KS, BS, and GM designed the study. YH and SH performed the experiments. YH and MH analyzed the data. YH drafted the manuscript. MH, BS, KS, and GM revised the manuscript. All authors have read and approved the final manuscript.

## Conflict of Interest Statement

The authors declare that the research was conducted in the absence of any commercial or financial relationships that could be construed as a potential conflict of interest.

## Footnotes

1 https://triticeaetoolbox.org/barley/view.php?table=line_records&uid=302

2 https://npgsweb.ars-grin.gov/gringlobal/accessiondetail.aspx?id=1288055

3 https://triticeaetoolbox.org/barley/

## Abbreviations

CIM: composite interval mapping
DON: deoxynivalenol
FHB: Fusarium head blight
HD: heading date
HT: plant height
KD: kernel discoloration
QTL: quantitative trait locus (loci)
RIL: recombinant inbred line
SD: spike density
SL: spike length
SNP: single nucleotide polymorphism
