# Supplementary material for: QTL Mapping of Fusarium Head Blight and Correlated Agromorphological Traits in an Elite Barley Cultivar Rasmusson
Source: Front Plant Sci. 2018 Aug 28;9:1260. doi: 10.3389/fpls.2018.01260 (PMC6127635; doi:10.3389/fpls.2018.01260)
Supplement: Supplementary file 1 [file Table_1.DOCX]

**QTL Mapping of Fusarium Head Blight and Correlated Agromorphological Traits in an elite barley cultivar Rasmusson**

Yadong Huang^1^, Matthew Haas^2,†^, Shane Heinen^1^, Brian J. Steffenson^2^, Kevin P. Smith^1^, and Gary J. Muehlbauer^1,3,*^

^1^ Department of Agronomy and Plant Genetics, University of Minnesota, St. Paul, MN, USA

^2^ Department of Plant Pathology, University of Minnesota, St. Paul, MN, USA

^3^ Department of Plant and Microbial Biology, University of Minnesota, St. Paul, MN, USA

^†^ Current address: Genebank Department, Domestication Genomics, Leibniz Institute of Plant Genetics and Crop Research (IPK), Corrensstraße 3, 06466 Gatersleben, Germany

^*^ Correspondence

Dr. Gary J. Muehlbauer

Muehl003@umn.edu

**Supplemental materials**

Figure S1. Scatterplot matrix of BLUP values combined over four environments for six traits. FHB, Fusarium head blight; DON, deoxynivalenol accumulation; HD, heading date; HT, plant height; SL, spike length; SD, spike density.

Figure S2. Genetic linkage map of 93 recombinant inbred lines (RIL) from the Rasmusson/PI383933 population based on 1,394 SNP markers. Genetic distance was calculated based on the Kosambi mapping function.

Table S1. Correlation coefficients among traits measured in four environments for the Rasmusson x PI383933 population. FHB, Fusarium head blight; DON, deoxynivalenol accumulation; HD, heading date; HT, plant height; SL, spike length; SD, spike density.
