# Supplementary figures and images for: QTL Mapping of Fusarium Head Blight and Correlated Agromorphological Traits in an Elite Barley Cultivar Rasmusson
Source: Front Plant Sci. 2018 Aug 28;9:1260. doi: 10.3389/fpls.2018.01260 (PMC6127635; doi:10.3389/fpls.2018.01260)

cM    Chr1H

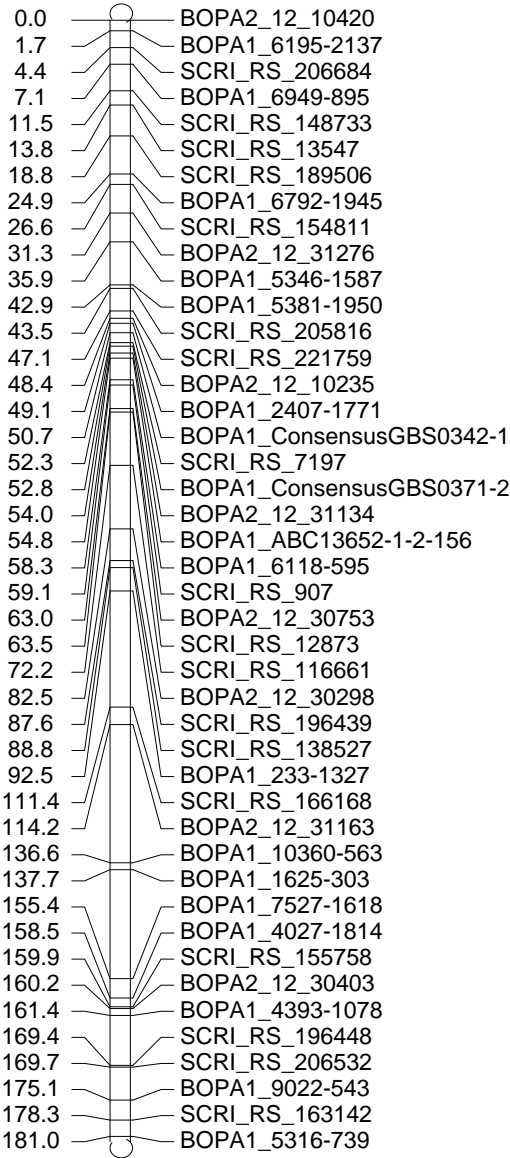

cM    Chr2H

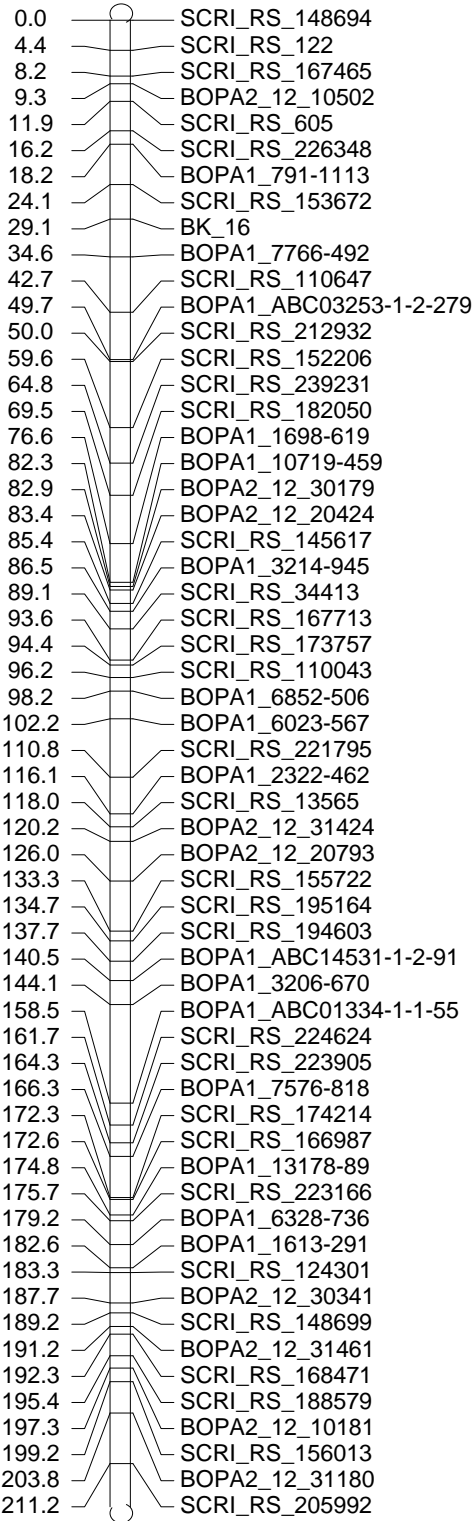

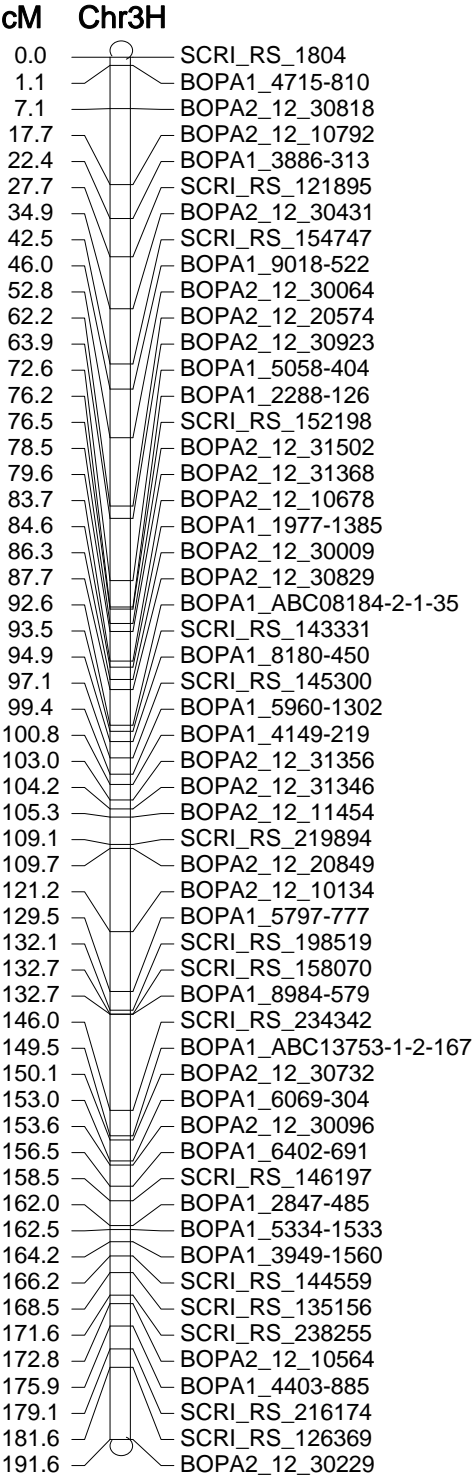

cM    Chr4H

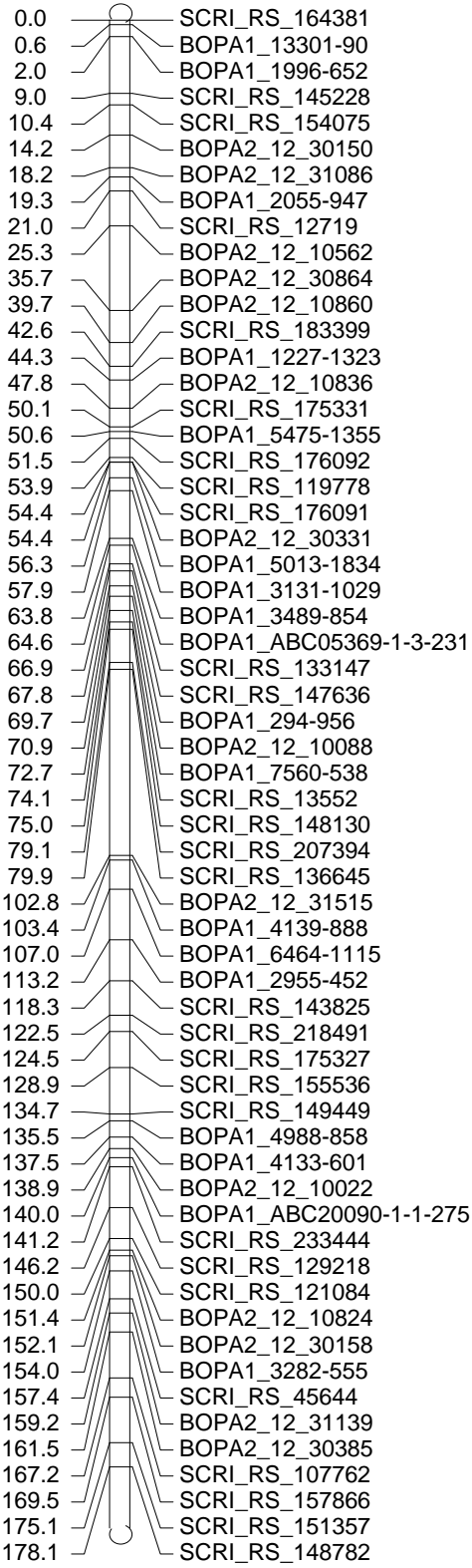

cM      Chr5H

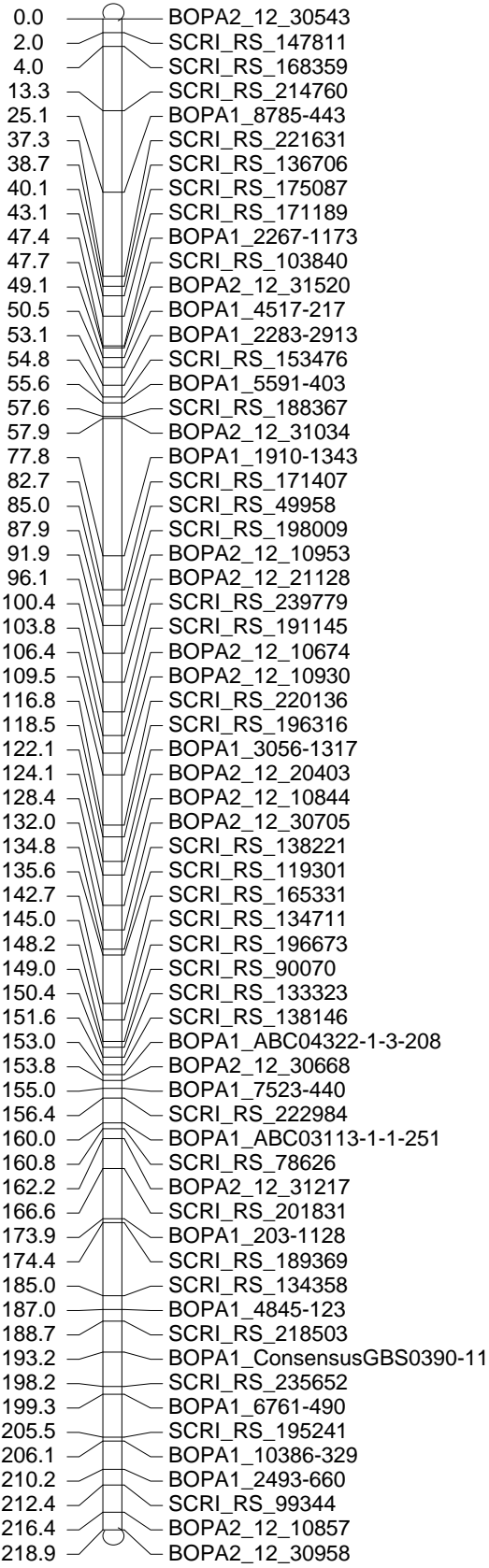

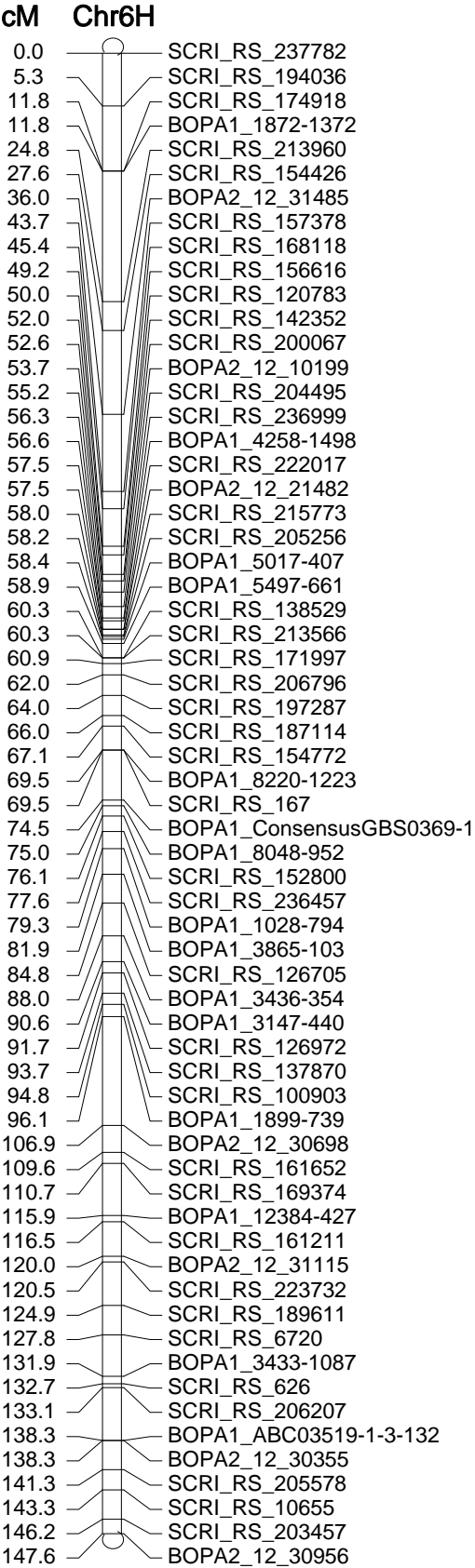

cM    Chr7H

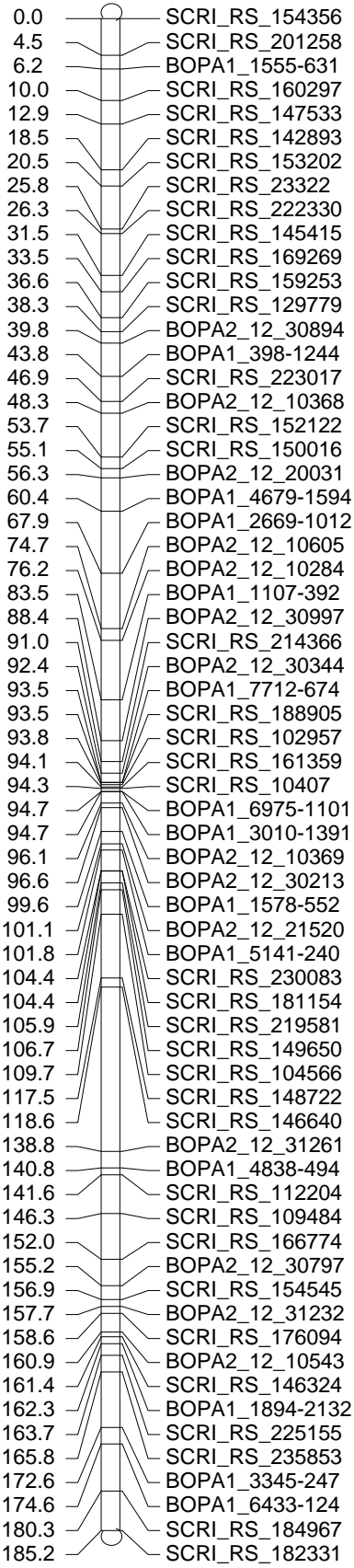

Supplement: Supplementary file 3 [file Data_Sheet_1.PDF]

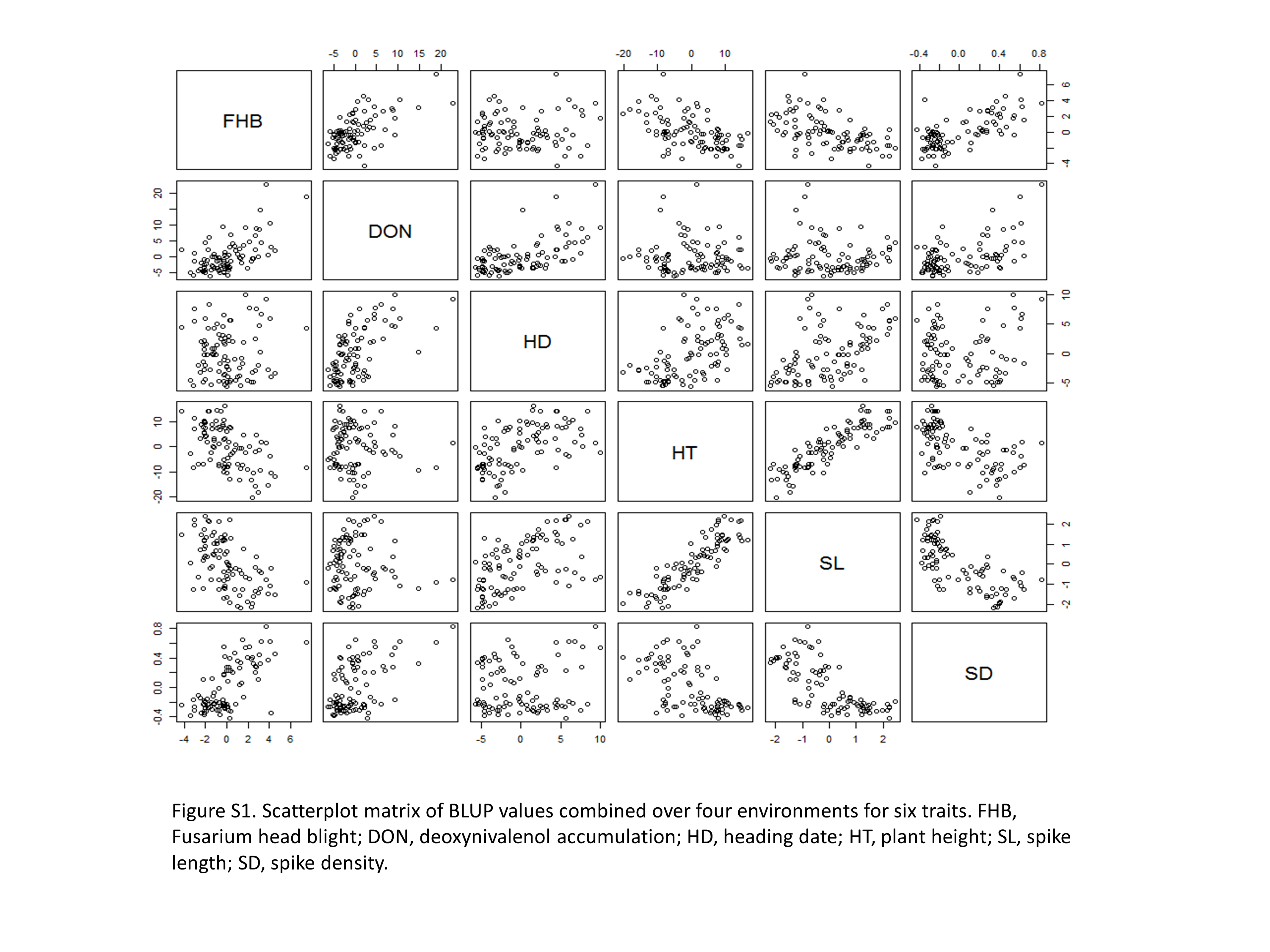

Supplement: Supplementary file 4 [file Image_1.TIF]
